# Advanced Strategies for the Fabrication of Multi-Material Anatomical Models of Complex Pediatric Oncologic Cases

**DOI:** 10.3390/bioengineering11010031

**Published:** 2023-12-27

**Authors:** Arnau Valls-Esteve, Aitor Tejo-Otero, Núria Adell-Gómez, Pamela Lustig-Gainza, Felip Fenollosa-Artés, Irene Buj-Corral, Josep Rubio-Palau, Josep Munuera, Lucas Krauel

**Affiliations:** 1 Innovation Department, SJD Barcelona Children’s Hospital, Santa Rosa 39-57, 08950 Esplugues de Llobregat, Spain; 2 Medicina i Recerca Translacional, Facultat de Medicina i Ciències de la Salut, Universitat de Barcelona, 08007 Barcelona, Spain; 3 3D Unit (3D4H), SJD Barcelona Children’s Hospital, Santa Rosa 39-57, 08950 Esplugues de Llobregat, Spain; 4 Centre CIM, Universitat Politècnica de Catalunya (CIM UPC), Carrer de Llorens i Artigas, 12, 08028 Barcelona, Spain; 5 Department of Mechanical Engineering, Barcelona School of Industrial Engineering (ETSEIB), Universitat Politècnica de Catalunya, Av. Diagonal, 647, 08028 Barcelona, Spain; 6 Pediatric Surgical Oncology, Pediatric Surgery Department, SJD Barcelona Children’s Hospital, Universitat de Barcelona, 08950 Barcelona, Spain; 7 Maxillofacial Unit, Department of Pediatric Surgery, Pediatric Surgical Oncology, SJD Barcelona Children’s Hospital, Universitat de Barcelona, 08950 Barcelona, Spain; 8 Diagnostic Imaging Department, Hospital de la Santa Creu i Sant Pau, 08027 Barcelona, Spain; 9 Advanced Medical Imaging, Artificial Intelligence, and Imaging-Guided Therapy Research Group, Institut de Recerca Sant Pau—Centre CERCA, 08041 Barcelona, Spain

**Keywords:** additive manufacturing, surgical planning prototypes, fused deposition modelling, fused filament fabrication, indirect 3D printing, selective laser sintering, material jetting, oncology, surgery, complex oncological cases

## Abstract

The printing and manufacturing of anatomical 3D models has gained popularity in complex surgical cases for surgical planning, simulation and training, the evaluation of anatomical relations, medical device testing and patient–professional communication. 3D models provide the haptic feedback that Virtual or Augmented Reality (VR/AR) cannot provide. However, there are many technologies and strategies for the production of 3D models. Therefore, the aim of the present study is to show and compare eight different strategies for the manufacture of surgical planning and training prototypes. The eight strategies for creating complex abdominal oncological anatomical models, based on eight common pediatric oncological cases, were developed using four common technologies (stereolithography (SLA), selectie laser sinterning (SLS), fused filament fabrication (FFF) and material jetting (MJ)) along with indirect and hybrid 3D printing methods. Nine materials were selected for their properties, with the final models assessed for application suitability, production time, viscoelastic mechanical properties (shore hardness and elastic modulus) and cost. The manufacturing and post-processing of each strategy is assessed, with times ranging from 12 h (FFF) to 61 h (hybridization of FFF and SLS), as labor times differ significantly. Cost per model variation is also significant, ranging from EUR 80 (FFF) to EUR 600 (MJ). The main limitation is the mimicry of physiological properties. Viscoelastic properties and the combination of materials, colors and textures are also substantially different according to the strategy and the intended use. It was concluded that MJ is the best overall option, although its use in hospitals is limited due to its cost. Consequently, indirect 3D printing could be a solid and cheaper alternative.

## 1. Introduction

Surgical planning is the process carried out by surgeons to predefine the surgical steps and approaches to be taken before surgery. Preparing and following the right steps can make a huge difference to the outcome of surgery, while poor preparation might lead to undesired complications. Surgical planning is carried out in most cases with the use of imaging diagnostics and analysis of DICOM (Digital Imaging and Communications in Medicine) data, usually from a CT (Computer Tomography) or an MRI (Magnetic Resonance Imaging). CT is used for obtaining detailed internal images of the body, which depend on the measure of X-ray attenuations by different tissues inside the body. MRI is a non-invasive technique that uses nuclear magnetic resonance to obtain information about the structure and composition of the body to be analyzed. CT is preferred for fast scanning of bones and skeletal structures, but MRI is more accurate for soft tissues and the analysis of certain diseases. Although this approach is the gold standard, in some cases, 3D visualizations are needed for a better spatial understanding of the anatomical relations of the case. Therefore, there are 3D reconstruction, projection and advanced visualization techniques, such as Cinematic Rendering or Volume Rendering, which allow for a photo-realistic and 3D visualization of the anatomical parts [1]. However, these visualization techniques are very limited in facilitating the simulation of surgery or the design of support tools.

To overcome these problems, doctors in different hospitals around the world are starting to use VR (Virtual Reality) techniques, in which 3D models can be seen in an immersive setting [2,3]. It allows surgeons to better understand the tumor-related anatomical structures and plan the best surgical approach. This also allows interaction with the 3D anatomy and simulates the surgical procedure.

Another approach to surgical planning is the manufacture of patient-specific 3D-printed devices or anatomical models, which can be used by surgeons for hands-on training, such as surgical aid tools or to enhance patient comprehension of the pathology. These tools have demonstrated their clinical effectiveness [4]. To develop realistic models, the use of the latest technologies, such as Additive Manufacturing (AM), is necessary.

The last-mentioned term, also commonly known as 3D printing, is the process by which a sample is manufactured layer-by-layer [5], as opposed to some traditional technologies in which, for example, different parts are removed from a solid block in machining processes. AM technologies are revolutionizing manufacturing industries by enabling the development of devices and products at the point of demand in a unique way. There are seven categories of AM technologies according to ISO/ASTM 52900 [6]: (1) Vat Photopolymerization (VP), (2) Material Extrusion (ME), (3) Material Jetting (MJ), (4) Binder Jetting (BJ), (5) Powder Bed Fusion (PBF), (6) Directed Energy Deposition (DED), and (7) Sheet Lamination.

Previous research studies show different approaches in the use of 3D printing technologies for the manufacture of surgical planning prototypes [7]: (1) the use of fused filament fabrication (FFF) technology (ME category), (2) using selective laser sintering (SLS) printing technique (PBF category), (3) employing the indirect 3D printing technique (different categories applied), (4) and the use of stereolithography (SLA) technology (VP category) or (5) Material Jetting technology (MJ). All these technologies show different advantages and disadvantages, which will be highlighted in the present paper.

On the other hand, these 3D-printed prototypes may also be used for patient education [8,9] or undergraduate or residents’ medical training [10,11]. These new technologies have been making their way into point-of-care settings in recent years [12,13,14].

The aim of the present study is to show eight different alternatives for the manufacture of complex oncological surgical planning prototypes depending on the budget, time and complexity of the procedure. Therefore, different AM technologies will be used to manufacture 3D-printed pediatric oncological models, showing the advantages and disadvantages of each option. Additionally, the time and costs of each option are summarized.

## 2. Materials and Methods

This work studies eight 3D printing and manufacturing strategies for the production of complex oncological anatomical models for use in surgical planning and as surgical training tools. Four of the most common AM technologies are being used at the point-of-care: SLA, SLS, FFF and MJ, plus indirect 3D printing and the hybridization of 3D printing techniques. In terms of materials, nine of them were selected due to their properties. Final models were compared in terms of application properties, production time and cost.

### 2.1. 3D Printing Technologies

#### 2.1.1. Fused Filament Fabrication (FFF)

Fused Filament Fabrication, also known as Fused Deposition Modelling (FDM), is the process in which a thermoplastic material is deposited onto a hot plate layer-by-layer. The most common material is PLA (Polylactic Acid) since it is hard and easy to 3D print. However, if other properties are required, such as support material or flexible parts, then PVA (Polyvinyl Alcohol) and TPU (Thermoplastic Polyurethane), respectively, are used. Using PVA, supports can be removed by placing the 3D model under water if a soluble support has been used. Otherwise, the support parts have to be removed manually.

**Figure 1:**
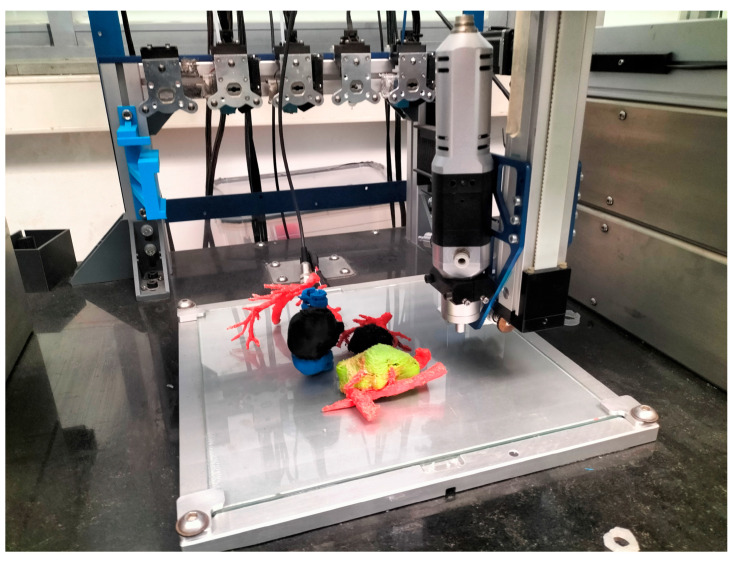
Multi-material “QuirofAM Project” 3D printer developed at CIM UPC under HSJD design specifications, combining FFF and Direct Ink Writing (DIW) technologies to advance on AM anatomical models [14].

The 3D printers used in the study are: (1) BCN3D Sigma R19 (BCN3D, Barcelona, Spain) and (2) customized 3D printer (see Figure 1).

#### 2.1.2. Selective Laser Sintering (SLS)

SLS technology is part of the PBF (Power Bed Fusion) category. SLS is focused on a laser beam that goes through the surface of a polymer powder by successively solidifying different layers of material. The material is heated slightly below the melting temperature and solidification occurs due to the effect of a laser beam, which causes heating above the sintering temperature. This happens when grain viscosity decreases with temperature, causing superficial lesions which, without merging, generate an interfacial union between the grains. Then, dust particles unaffected by the laser beam remain unbonded and act as support material. The platform then goes down a layer, and the process starts again, until the model is finished. Once the printing is over, SLS technology has a cooling down process that lasts approximately 12 h, which prevents possible thermal distortions. After the cooling process, the entire material batch is taken out and the remaining (unsintered) material is removed by a manual process that takes approximately 40 min. Once the printed part is removed, a quick sand blasting operation is necessary to remove the powder. Other post-processing techniques can be used if necessary. For instance, models can be colored after sand blasting.

The SLS 3D printer used is Ricoh AM S5500P (RICOH, Tokyo, Japan).

#### 2.1.3. Material Jetting (MJ)

MJ is based on the jetting of ultra-thin layered photopolymers on a build deck. Each layer of photopolymer cures immediately after spraying with UV light, allowing fully cured products, which can be handled and used immediately, to be generated without the need of a post-process phase. Gel-like support, designed to allow the construction of complex geometries, is subsequently removed by jets of water.

The 3D printers used for the present study are Connex 3 and J5 MediJet (Stratasys, Eden Prairie, MN, USA).

#### 2.1.4. Stereolithography (SLA)

SLA, which is included in the vat photopolymerization group, is one of the oldest 3D printing technologies known. It is based on the use of a UV laser to cross-link chemical monomers and oligomers from a tank of photopolymer resin to form polymers, thereby making up, layer-by-layer, the body of the three-dimensional solid. Once finished, the new 3D object needs to be post-processed. First, it has to be washed with a solvent to clean up the remaining liquid resin on its surface. Then, it has to be cured for several minutes in UV light to finish the solidification process.

The 3D printer used for the present study is Form 3BL (Formlabs, Somerville, MA, USA).

#### 2.1.5. Indirect 3D Printing

Indirect 3D printing is a process based on the manufacture of an outer 3D-printed mold using the negative of corresponding segmented tissue. The internal parts of the anatomy can be 3D printed separately and then placed inside the mold prior to the casting procedure. Despite being much more time consuming, this approach provides the opportunity to cast a soft material in the mold in order to mimic the mechanical properties of a softer tissue. The outer mold can be manufactured using either FFF or SLS technologies. This process requires the use of CAD programs to design the mold. Post-processing depends on the selected technology.

#### 2.1.6. Hybridization of AM Technologies

The hybridization of AM technologies is based on the manufacture of different parts of a surgical model using different AM technologies. In the present study, a combination of FFF and SLS parts is proposed. The SLS part is for harder pieces, while FFF parts are 3D printed using both PLA (hard parts) and thermoplastic polyurethane TPU (flexible parts).

### 2.2. Materials

Table 1 shows the 3D printing materials used in this study for each technology, as well as the manufacturer and country of origin.

#### 2.2.1. FFF

In this article, the materials used are PLA, PVA and TPU. PLA is the most widely used material in FFF technology. It is used mainly for manufacturing harder parts and visualization models, whereas TPU is mainly used for flexible parts. The TPU used in this case has a shore hardness of 60A. PVA is a support material typically used because of its easy post-processing in dual extrusion FDM printers, as it melts in water.

PVA and PLA were purchased from BCN3D Technologies (Barcelona, Spain) and TPU from Recreus Filaflex (Alicante, Spain).

#### 2.2.2. SLS

The material used is polyamide 12 (PA12), which is a hard and biocompatible material; therefore, it can also be used for the manufacture of surgical guides and surgical tools. It is a hard material with good properties of resistance to abrasion by chemicals, temperature or pressure. It can be sterilized with the main sterilization processes available in hospitals (autoclave sterilization).

The PA12 used is from 3D Systems (Rock Hill, SC, USA).

#### 2.2.3. MJ

The materials used are, on one hand, Vero White, an opaque and rigid photopolymer printed with the Connex 3 PolyJet printer from Stratasys, and SUP706, a gel-like, water-soluble, support material. On the other hand, the following materials were printed with the J5 MediJet printer from Stratasys: the hard, opaque and colorful photopolymers Vero Magenta and Vero Cyan; the elastic and translucent Elastico Clear photopolymer; and the gel-like, water-soluble, SUP710 support material.

All materials used are from the same manufacturer, Stratasys.

#### 2.2.4. SLA

For the manufacture of models using SLA technology, Surgical Guide Resin was used. It is a hard, translucent, biocompatible and serializable resin usually used for the manufacture of dental tools and surgical guides. This material is from the manufacturer Formlabs.

### 2.3. 3D printing Software

The CAD software used in this project was Materialise Mimics version 25 (Leuven, Belgium) for segmentations and anatomical model design and preparation, and Autodesk Meshmixer 3.5 (San Francisco, CA, USA) for design of the linking parts of the anatomical models in the hybridization strategy (7) and for the design of the casting mold (in strategy 8).

In the case of printing software, each company usually supplies its own slicing software. In the specific case of FFF, two different software programs have been used. Stratos is the software used by BCN3D printers, and the Simplify3D 4.1.0 software was selected for the customized FFF 3D printer because it allows a much higher degree of control and customization. For instance, Simplify3D allows the accurate control of more than two printheads and has a very useful scripting tool for experimental prints. Thus, Simplify3D was the best option for the case of the multi-material FFF strategy, in which a custom-made printer was used. Table 2 shows the design and 3D printing software used in each case depending on each technology, ordered according to the strategy of the present study.

### 2.4. 3D Printing Time

The 3D printing time is calculated considering the printing time plus the time used for post-processing. Each AM technology and material has its own post-processing process, which can modify the overall finishing time of the model. The time needed by the technical personnel dedicated to each model is also posted, as it can vary between technologies and could be a key decision factor. Price per hour is not included as it may vary a lot between institutions.

### 2.5. 3D Printing Costs

The 3D printing costs are calculated as the material cost per g or l of material used and the estimation of the fungibles needed for each technology. Material and fungible costs are taken from each manufacturer, most of which are publicly available.

## 3. Results

### 3.1. 3D-Printed Realistic Models

**Figure 2:**
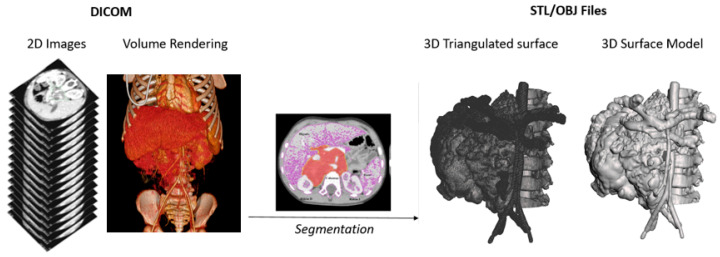
Schematic representation of the process of segmentation from DICOM imaging to a 3D surface triangulated file (STL—Standard Tessellation Language) and 3D model in OBJ format.

All models were obtained from the process of segmenting DICOM images obtained from CT Scan or MRI. This process transforms the sequence of 2D images into a 3D computer-aided design (CAD) surface object in STL or OBJ formats (as schematically explained in Figure 2). This process is carried out automatically or semi-automatically with the aid of specialized software. In this work, Materialise Mimics v.25 and Philips IntelliSpace Portal v.10 (Amsterdam, The Netherlands) software were used. This process is performed to obtain a CAD object to work with, and from which a 3D printed model could be created. It is not possible to develop the same manipulation and design as in CAD objects from an image representation or projection, such as Volume Rendering, since it is not a volumetric object, but a visualization effect.

Once the 3D file is obtained, it can be modified using CAD software (Autodesk Meshmixer for design) to obtain the desired final anatomical models and be prepared for printing using each manufacturer’s specific printing software (see Table 2).

**Figure 3:**
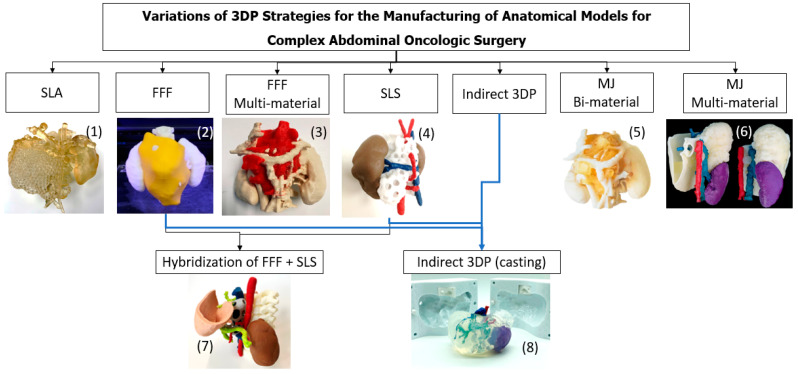
Summary of the different technologies ((1) SLA, (2) FFF, (3) FFF Multi-material, (4) SLS, (5) MJ Bi-material, (6) MJ Multi-material, (7) Hybridization) and (8) Indirect 3D Printing and anatomical models’ production strategies followed in the present work.

The 3D-printed anatomical models produced are the following, as summarized in Figure 3:

#### 3.1.1. SLA

**Figure 4:**
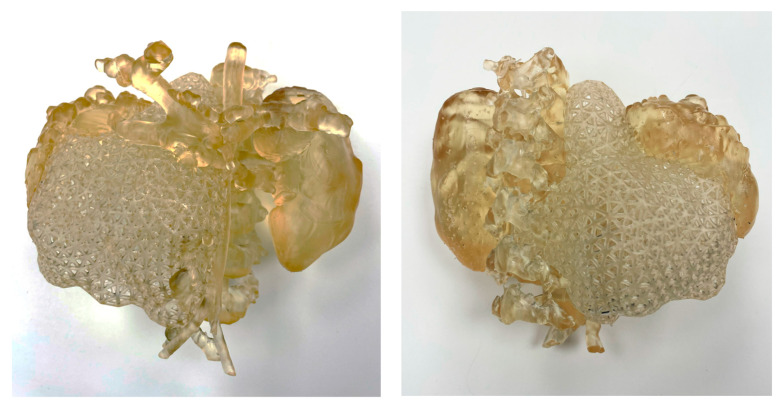
SLA 3D-printed model showing the anatomical parts in translucent magenta (tumor, vessels, spine and kidneys).

Figure 4 shows the 3D model, printed using an SLA 3D printer. This model is monocolor and mono-material printed with Surgical Guide Resin (Form 3BL, Formlabs). As this model is mono-material, the tumor was printed with a specific lattice consisting of a predefined mesh triangle. This allowed differentiating between the tumor and the vessels, kidney and spine as they had different textures. Support material was also printed with the same material as the model, which can create some challenges when post-processing the model to extract the support material from internal cavities. The post-process of the model involved washing it with isopropyl alcohol for 20 min to remove uncured resin from its surface, followed by post-curing for 30 min at 60 °C. Once the model was post-cured, the support material was removed manually.

#### 3.1.2. FFF

**Figure 5:**
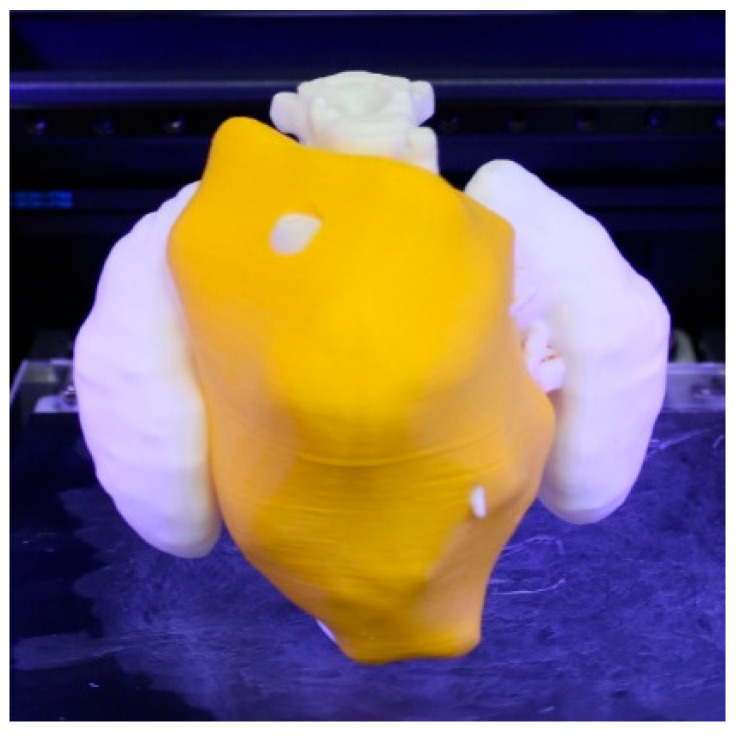
3D-printed realistic model showing the neuroblastoma in yellow and the rest of the anatomical structures (spine and kidneys) in white.

Figure 5 shows the 3D-printed model using a bi-material 3D printer (Sigma R19 BCN3D), using the same PLA material in different colors (white and yellow) and the same PLA material as a support, as a DUAL extrusion printer was used. The 3D printer used is an independent dual extruder printer, which shows better performance compared to other mono-extruder 3D printers when at least one anatomical part needs to be differentiated from the rest of the anatomy. In this case, the neuroblastoma is differentiated in yellow. Interior encased vessels cannot be visualized.

Once the model was printed, it was post-processed by manually removing the support structures generated during the printing process.

#### 3.1.3. FFF Multi-Material

**Figure 6:**
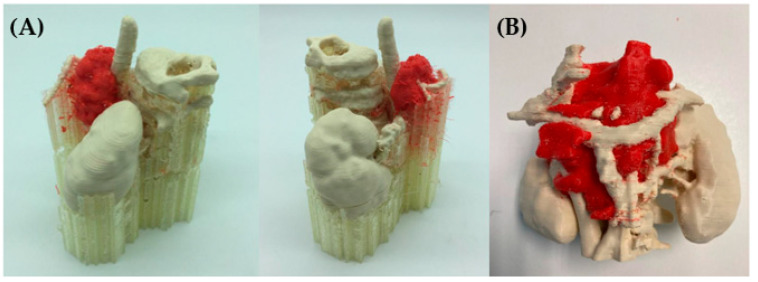
(**A**) 3D-printed realistic model showing the neuroblastoma in red in TPU and the rest of the anatomical structures (spine, vessels and kidneys) in white PLA. The other material is PVA, which is used for the support parts. (**B**) After the removal of the PVA, the realistic 3D-printed model is visualized [14].

Figure 6 shows the realistic 3D models, printed using a multi-material 3D printer. Unlike the previous model, in this technology a third extruder was used. The machine used was a self-developed FFF multi-extruder printer, shown in Figure 1. Having a third extruder provides the option of using PVA as support material instead of using one of the constitutive model materials, which facilitates the removal of scaffolding backing material in post-processing, as in the case depicted in Figure 5. In this model, the tumor is differentiated in red and printed in TPU soft material to obtain a better mimicry of the tissue. The spine, kidneys and vessels were printed using PLA.

After the realistic model was manufactured, the majority of the support material was removed manually and then the prototype was immersed in warm water for approximately 4 h so that the remaining PVA material was fully removed.

It must be mentioned that the multi-material equipment used in this study is an experimental printer. Therefore, the optimization of the printing times has not yet been carried out. For instance, almost 40% of the printing time corresponds to the heating time of the printheads produced between every tool change. In commercial equipment, the heating of the printhead starts before the tool change.

#### 3.1.4. SLS

**Figure 7:**
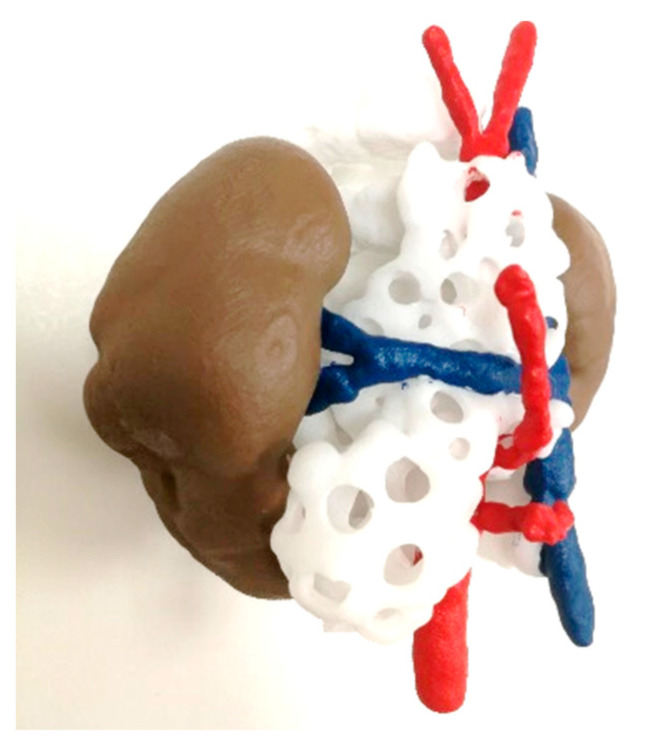
Realistic 3D-printed model painted after post-processing, showing the neuroblastoma in white, the kidneys in brown, the veins in blue and arteries in red.

Figure 7 shows a 3D model printed on an SLS 3D printer (RICOH AM S5500P) and using PA12 material. Unlike the previous three models, with the present model, it is necessary to paint the different anatomical structures in order to highlight each tissue. Painting is carried out as a post-process, after printing the anatomical model in white and sand-blasting.

A remarkable feature about this technology is that it does not require support material, since the uncured powder itself acts as a support. Most SLS printers have big build volume so that different models can be manufactured at once. However, it has the drawback that the post-processing takes time, as it includes different steps such as waiting for the piece to cool down (~12 h), manually removing all the remaining powder with a sand blaster, and painting the model by hand.

Regarding the holes in the tumor (white part); they were made by design in order to have visual access to the tumor, providing a better anatomical relation assessment and allowing other important anatomical structures such as the blood vessels inside the tumor to be seen.

#### 3.1.5. Hybridization of FFF and SLS

**Figure 8:**
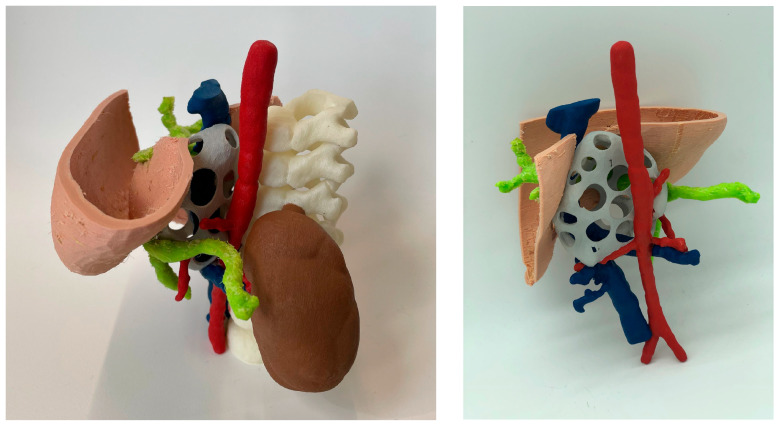
Realistic 3D-printed model obtained by the hybridization of FFF and SLS technologies. The spine (white), blood vessels (blue and red) and kidneys (brown) structures were manufactured using SLS. On the other hand, liver (flesh) and portal system (green) were manufactured using FFF in TPU soft material.

Figure 8 shows the models that were 3D printed using a combination of two different AM technologies (FFF and SLS). This hybridization allows complex prototypes that combine hard and soft materials to be developed, providing a more realistic approach by giving the surgeon a better view and approximation of the surgery.

However, it is time-consuming to print, as two different techniques are needed. The printing time of the SLS part (using a RICOH AM S5500P machine and PA12 material) took 12 h, while the FFF model (using a R19 Sigma BCN3D printer and a combination of PLA and PVA as support material) took 26 h.

For the FFF model, the post-process consisted of PVA support removal, first manually, and then the remaining material was removed by immersing the model in warm water (~4 h). Regarding the SLS part, the post-processing procedures involved waiting for the model to cool down (~12 h), manually removing the support material and painting the different colored parts by hand.

#### 3.1.6. Indirect 3D Printing

**Figure 9:**
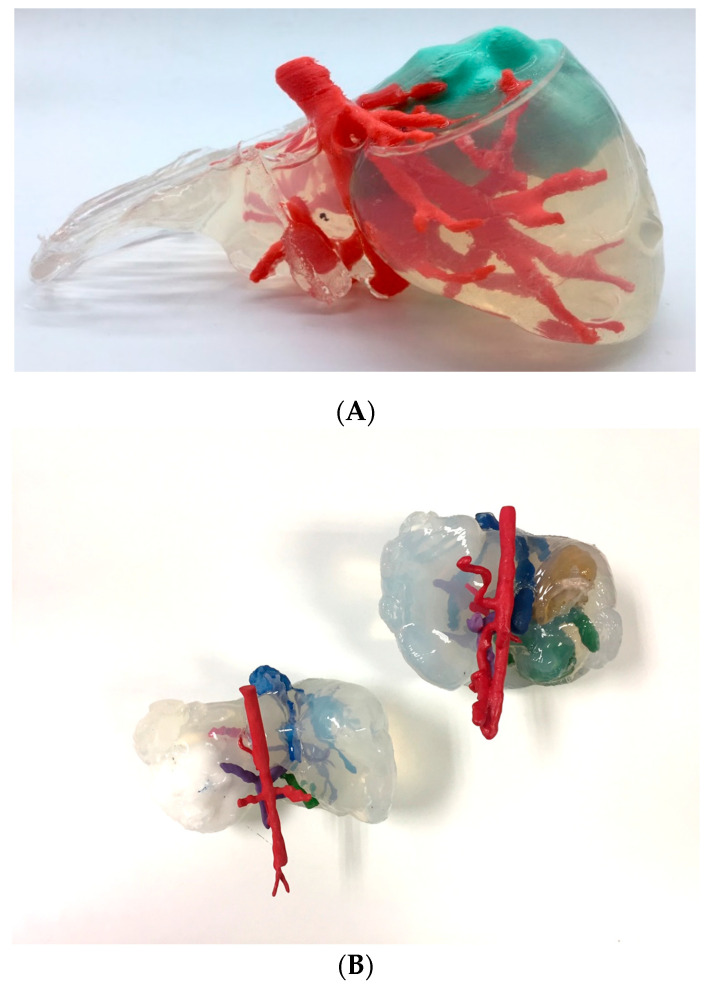
Realistic 3D-printed models produced by indirect 3D printing, with blood vessels and tumor in flexible TPU (**A**) and casting agarose hydrogel (Químics Dalmau, Spain), (**B**) with vessels, biliary tract and tumor produced by SLS, painted in different colors, and casting Dragon Skin^®^ silicone for the liver.

Figure 9 shows two alternatives to the models produced using the indirect 3D printing approach. Figure 9A shows a case of hepatic tumor. Being hepatic metastasis, the anatomy and AM strategy could be applied to many tumors, as well as neuroblastomas. Both blood vessels (red) and tumor (light blue) were manufactured using the TPU filament and 3D printed using the FFF technology (~12 h) on an R19 Sigma BCN3D printer. Regarding the mold, PLA filament was used for its manufacture using FFF (~18 h) with the same R19 Sigma BCN3D printer. Then, an agarose (agar-agar hydrogel, Químics Dalmau, Spain) solution mixed at 80 °C with H_2_O was cast and left to solidify (2 h). Figure 9B shows vessels printed using SLS technology (~12 h for printing and a further 12 to cool down) using a RICOH machine (AM S5500P) and painting during post-processing. Regarding the mold, PLA filament was used for its manufacture, using FFF R19 Sigma BCN3D (~18 h). Finally, soft silicone was cast and left until it was hardened (~4 h). This method is very attractive because it allows models that mimic soft tissues to be obtained, but it is more time consuming than other direct AM methods, since it is necessary to design and manufacture the molds, post-process the molds with varnish to make the silicone transparent (~2 h) and cast the silicone (~1 h).

The main advantage is that soft Dragon Skin^®^ silicone by Smooth On (Macungie, PA, USA) or hydrogels such as agarose offer the surgeon haptic feedback and a texture similar to living tissue, allowing for training with real surgical instruments.

#### 3.1.7. Material Jetting

**Figure 10:**
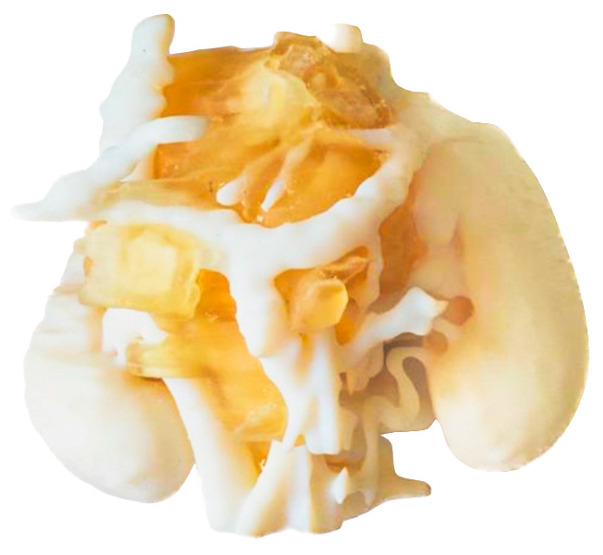
3D-printed model of a sarcoma manufactured by MJ using Vero and enclosed support material. Blood vessels, hearth and bronchioles are in Vero White, while the sarcoma tumor is translucent Tango Grey material.

Figure 10 shows the 3D models printed using a bi-material jetting 3D printer (Connex 3 Stratasys MJ printer) and Vero White resin and SUP710 support material. In this case, the support material used was enclosed in a thin 0.4 mm mesh of Vero material to generate the tumor part. The post-process of the model consisted of removing the soluble support material. The extraction of the support material had to be performed with a bath of hot water, with great care being taken to prevent the tumor enclosure from breaking.

This technique allows the manipulation of the anatomical model, providing the surgeons with an opportunity to simulate the tumor extraction operation if needed. The gel-like behavior of the support material can provide a quasi-realistic haptic sensation.

It is important to take into account that this model cannot be sterilized, as neither the support material nor the Vero material are biocompatible; thus, it can only be used for training or visualization purposes and cannot be used in the operating room.

#### 3.1.8. Multi-Material Jetting

**Figure 11:**
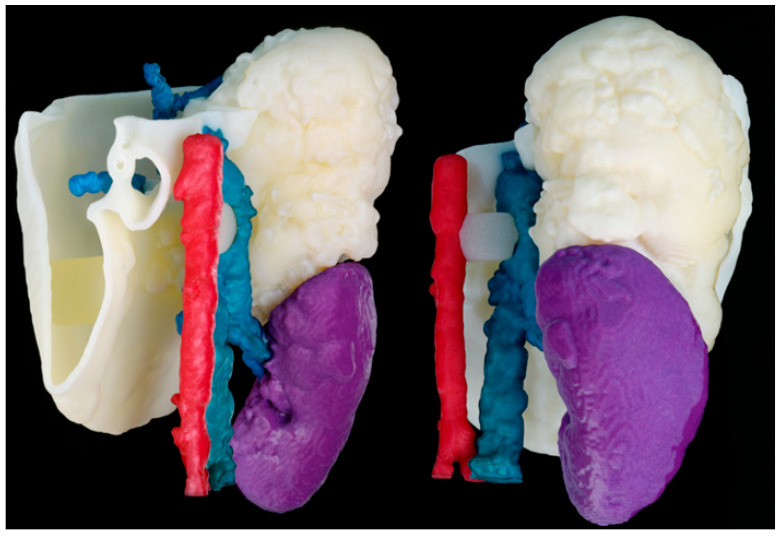
3D-printed realistic model MJ multi-material. Veins are in blue, arteries in red, kidney in purple, the tumor in light yellow and the liver in soft white material.

Figure 11 shows the realistic 3D models printed using an MJ multi-material 3D printer (Stratasys J5 MediJet). This is a complete combination of Vero colorful resins, support gel material and elastic resin. This combination allows for highly realistic approaches with multiple-color and multiple-texture models with tumors in elastic material, and each anatomical part with different colors. This technology and approach also allows defining different shore hardness values for each anatomical part. The liver was printed with the elastic resin to give it more realism and the tumor was printed in a combination of Vero Yellow resin with elastic resin. Cylindrical support structures were manually placed and printed to ensure that all the anatomy was joined in the final 3D-printed model without losing its original location. The model was post-processed by soaking it for 24 h in a mixture of water, caustic soda and sodium metasilicate, then the support material was further manually removed using pressurized water.

### 3.2. 3D Printing and Processing Time

Table 3 summarizes a comparison of the 3D printing and post-processing times. This table also includes the time dedicated by technical personal in the total post-processing time, as well as the complexity of the post-processing (labor tasks) classified in a range from low to high. For example, FFF parts are not complicated to post-process and do not require as much post-processing as other techniques such as SLS and MJ. The only post-processing that takes place in FFF is the removal of support material (which can be water-soluble support such as PVA, requiring more time to remove but lower personnel time, and the normal support material which has to be eliminated manually). However, in SLS, it is necessary to cool down the final model, remove the powder material left and sand-polish the parts. This process calls for specialized personnel, manufacturing environment and facilities. In the case of SLA, post-processing requires the removal of support material after curing and washing with alcohol; this process may be slightly more complicated than FFF as it involves more steps and the support material is the same as that of the model, which requires extra polishing before releasing the product. Finally, in the case of indirect 3D printing, the labor complexity is the highest, as it requires mold preparation, casting, air removal, curing, demolding and surface finishing. As a summary, the complexity of post-processing can be classified as follows:

1. Low: FFF—remove support material. Soak in water or soda.
2. Medium: SLA—model curing, washing and removing support material.
3. Medium–High: SLS post-processing
4. High: Indirect 3D printing (casting, molding, etc.)

Table 3 provides a summary of the production and post production time required for the different strategies analyzed in this work. 

**Table 3:** 3D printing, processing time and complexity of each approach for the manufacture of 3D-printed surgical planning prototypes. * Time can vary depending on the dimensions of the model.

Strategy (Figure 3)	Technology	Production and 3D Printing Time *	Post-Processing Time (PpT) without Personnel	PpT Dedicated by Tech. Personnel	Labor Complexity	Total Time *
1	SLA	12 h–18 h	50 min	10 min	Medium	13 h–19 h
2	FFF	12 h–18 h	-	30 min	Low	12 h–18 h 30 min
3	Customized FFF printer	45–50 h	4 h	30 min	Low	49 h–54 h 30 min
4	SLS	12 h–18 h	12 h	1 h 30 min	Medium/High	25 h–30 h 30 min
5	MJ Bi-material	24 h–36 h	24 h	30 min	Medium	48 h–60 h 30 min
6	MJ Multi-material	24 h–36 h	24 h	30 min	Medium	48 h–60 h 30 min
7	Hybridization of FFF + SLS	38 h–45 h	16 h	2 h	Medium/High	56 h–61 h
8	Indirect 3D Printing (casting)	34 h–40 h	2 h	3 h	High	38 h–45 h

### 3.3. Costs

Table 4 summarizes a comparison of the manufacturing cost of the 3D-printed prototypes in each technical approach. The 3D printing material cost only considers the material used in each case. It does not count the personnel costs as they may vary between institutions. Table 4 also shows the cost of each machine as a key factor in the calculation of the final cost per piece and the cost of the fungibles for each 3D printing technology. Fungibles in SLA include the cost of the resin tank (which is one tank for each material type); in FFF, the fungibles include the replaceable extruders. In the case of SLS technology there are fungible parts (lens, joint maintenance and roller belts) that have a life expectancy that is similar to the amortization time of the machines; thus, this cost is included in the machine cost and not added as extra fungible cost. Finally, in PolyJet (MJ), the extruders are also fungibles, but, as opposed to FFF, in MJ it is not necessary to change them so frequently. If nothing unexpected occurs, their life expectancy is similar to the time needed for the machine amortization; thus, their cost is not significant when calculating the model cost.

### 3.4. Comparison of Material Properties

As can be seen from the above, there is a wide variety of materials and processing technologies. The material properties have a great impact on the final function of the model. However, the final aim in the present study is the manufacture of not only realistic models in terms of resolution, size, geometry, etc., but also in the mechanical behavior of the materials. In other words, achieving a match between the tissue and the 3D-printed materials’ haptic sensations.

Resolution is also a key factor in ensuring high-quality end parts. Each technology presents its own limitations in terms of resolution, measured as the minimum layer thickness possible. Table 5 summarizes each technology’s layer resolution in µm. Indirect 3D printing is limited by the resolution of the technology used for casting the molds, with the inner manufactured parts being FFF and SLS in the case presented in the current study.

## 4. Discussion

The aim of this study is to present a multi-approach for planning and training in complex oncological abdominal cases, such as neuroblastomas or hepatic tumors, with advanced multi-material 3D models using AM technologies. In light of the results, it is shown that each technique has its own advantages and disadvantages.

### 4.1. 3D Printing and Processing Time

Today, 3D printing technology cannot be considered a fully automatized production system, as it still requires some manual input, mainly when it comes to setting up the print, removing it from the machine and post-processing the models [15]. This last step can show significant variations between technologies in terms of the time consumed, as can be seen in the results obtained from the present study. Thus, when selecting the technologies to be used, it is important to take this time into account, and not only the printing time.

Amongst the different process mentioned, FFF + SLS is the longest one, due to the different steps needed in post-processing, especially waiting for the material to cool down after printing in SLS. Nevertheless, most of the post-processing procedures are semi-automated, which make the work less labor-intensive than other strategies, such as indirect 3D printing. Indirect 3D printing may combine at least two technologies in its production and the generation of molds for casting silicon or hydrogel materials such as agarose. This process requires trained technical personnel, while also requiring the greatest dedication of personnel in post-processing. Printing time is limited by the technologies used for casting and printing the cast anatomical parts [16].

Of all the technologies evaluated, SLA and FFF are the least time consuming. However, when it comes to post-processing time, it is important to differentiate the time consumed by labor from that automatically performed by a post-processing machine. In that regard, our results show a significant difference in the time needed for post-processing in FFF technology as compared to SLA, as the latter needs specific curing and washing machines to aid this process. The same occurs with the MJ technology, being one of the least labor-intensive of the technologies compared.

The FFF multi-material approach is based on a self-produced machine which is still in development [14]. It currently presents the highest printing times; however, almost 40% of the printing time corresponds to the heating time of the print heads between every tool change. In commercial equipment, the heating of the print head starts before the change of the tool. Thus, it is believed that, when fully developed, the printing time could be reduced by 30%, becoming similar to other commercial technologies.

Finally, when comparing the total time needed for the production of a model, taking into account the printing and post-processing time, MJ represents the most time-consuming, followed closely by the FFF and SLS combination and indirect 3D printing. Total production times are in line with other works [17].

### 4.2. Costs

In terms of costs, as can be seen in Table 4, SLS and MJ are the most expensive approaches. In the case of SLS, the machine amortization cost is very high and the material cost is placed in mid-range, but post-processing time needs significant human intervention, making the overall production cost one of the highest. In the case of MJ, the cost of material is higher than in other technologies, but the post-processing labor time is lower, being one of the most automated AM technologies. The machine cost is in mid-high-range, higher than SLA or FFF, but significantly lower than SLS.

Moreover, FFF technology is the most affordable option, with a low-cost 3D machine and with the lowest material cost. FFF 3D printing technology is cost-effective, and can be used as a good method for visualizing 3D models if high-accuracy finishing is not needed. SLA provides a slightly higher resolution compared to FFF, with the limitation of being able to print only one material at a time [18]. Thus, FFF represents a solid option for the manufacture of low-cost, patient-specific 3D printed anatomical models, as also discovered by other authors [19].

### 4.3. Properties and Applications

Firstly, it is important to highlight that all the strategies and 3DP/AM technologies presented for the manufacture of patient-specific 3D-printed anatomical models show a high level of dimensional accuracy. This is in accordance with other works [20].

Multi-material 3D prototypes are a better approach for the production of oncological anatomical models, in comparison with mono-material 3D technologies. Having different materials, with different mechanical properties and colors in the prototypes, makes each part easier to highlight and differentiate [21]. Following the presented strategies, this is not accomplished in the case of SLA technology, in which the need for a unique resin tank limits the ability to produce multi-material models in just one print. An alternative could be to split the manufacture of the model into multiple prints, all on the same machine but using different materials, and to finally join the resulting pieces. This procedure would increase the post-processing time, but allow for a multi-material model using only SLA technology. Another solution to this drawback is the application of textures to easily identify the different anatomical structures, as in the model presented in this article. MJ is the best approach when multi-color, multi-texture and multi-hardness are needed, as it allows the combination of multiple resins in the same print. This is also seen in other works [22,23].

The combination of two AM technologies for the manufacture of 3D models is not a common procedure, but it may be an option when different textures are needed for complex surgeries involving different anatomical parts [20]. Nevertheless, this can be a challenge in high time-demand environments such as hospitals, in which complex cases may require urgent surgery to be performed in less than 48 h.

Indirect 3D printing is one of the most cost-effective methods used in this field when multi-color and multiple hardness values are needed. This technique allows for the combination of textures and materials beyond the range of available materials in most 3D printers. Nowadays, indirect 3D printing strategies focus on the use of three different materials: (1) PVA and PHY (Phytagel) [24]; (2) agarose [25]; and (3) silicone [26,27,28]. These materials allow the improvement of final 3D models when specific softness, low-hardness values or certain viscosities are needed. Some have certain drawbacks, such as the case of agarose, since its gelatinous consistency makes it very fragile and difficult to work with. Moreover, agar-agar has good hardness properties when compared to liver tissue; however, its main limitation is the degradation of the model in approximately 48 h after production, due to its high H_2_O content [25].

There are some limitations in each approach. For example, FFF, SLA and SLS are mainly useful for visualizing anatomical structures or for a fast-printed model. These approaches could be useful for patient–professional communication, especially with SLA and FFF technologies that provide the cheapest options. SLS, while more expensive overall and requiring long post-processing times, is highly accurate and has the ability to print multiple models on a single tray, reducing the final cost and having few geometrical limitations. This can be a good alternative for surgical tools and educational purposes, and it could be colored in the post-processing phase. A similar situation is presented with the indirect 3D printing approach. These results are in line with the work of Yang et al. [29]. This strategy takes a lot of labor effort, which might not be the most suitable for surgical planning in time-restricted situations. Nevertheless, it could be a good alternative for simulation and educational purposes.

Another important factor to be taken into account in biomedical applications is the ability of materials and end parts to withstand sterilization processes (chemical or physical) and the certification and validation of those materials to withstand such processes. Depending on their final application, and whether they must enter sterile environments such as operating theaters, the anatomical models produced will need to be sterilized. Materials and technologies such as MJ with its medical grade materials such as MED610 (a hard, translucent resin photopolymer), SLS with its medical grade material PA12 or SLA with Surgical Guide Resin are better prepared and validated for medical use when sterilization is needed [30].

Last but not least, jetted photopolymer, a Material Jetting technology, was the most promising option [31]; it allows for multi-color, multi-texture final models with just one print, combining soft and hard tissues and presenting the highest accuracy of all the technologies studied. Moreover, nowadays a wide range of medical grade MJ materials and technologies are available on the market. Although it seems to be the most robust solution, the high cost limits its use in hospitals, especially in the public sector, with it only being affordable for specific mature point-of-care 3D units or hospitals with a sufficient budget.

### 4.4. Mechanical Properties: Seeking to Mimic Real Tissue

Huge efforts have been made in the field of materials for the 3D printing of realistic biomedical anatomical models in terms of mechanical properties, not only by the authors of the present study but also by other researchers, which can be summarized for a more in-depth analysis in the following studies [32,33,34].

For instance, nowadays the gold standard for surgical training continues to be live animals or cadaveric specimens of human or animal origin. However, it is known that post-mortem degradation and the use of preservation methods have an effect on the mechanical properties of tissue [35]. Moreover, the amount of time a cadaveric specimen can be used is limited by postmortem time [36,37]. Although there is still a lack of data to define an exact degradation time for cadaveric specimens, most of the studies suggest they start to degrade after 36 to 72 h [36]. Thus, synthetic anatomical models represent a promising alternative, allowing models to be preserved for longer, while at the same time costing less [38]. The main challenge is to ensure proper mimicry of tissue biomechanics.

On the one hand, the elastic modulus of the materials used with FFF, SLS and MJ are in the range of the MPa, which are close to hard tissues such as bone [39,40,41]. For instance, PLA from FFF, PA12 from SLS and VERO from MJ have an elastic moduli of 1568 ± 45 MPa, 1487 ± 48 MPa and 1492 ± 175 MPa respectively [30]. However, these materials do not match soft tissues such as the liver or heart, because their elastic modulus is in the range of kPa [33,34]. In this case, the anatomical models produced with those materials are mainly used for surgical training, visualization, patient–professional communication, surgical planning and last minute check approaches: looking for anatomical references, size of tumors, implant matching and anatomical relations [42].

On the other hand, hydrogels or silicones are used to mimic soft tissues. They represent a promising alternative to polymeric and synthetic approaches, as they are the best for achieving the softness and stiffness of soft tissues such as liver, kidney, tumor, vessels and others, and have mimicking qualities, since the range of kPa can be obtained [43]. Tejo-Otero et al. [25] measured an elastic modulus of 5.5 kPa for the 1%wt agarose and 18.1 kPa for the 4% GelMA. This kind of soft material can be cast in 3D-printed molds [44]. For example, Tejo-Otero et al. [25] developed a biliary tract rhabdomyosarcoma liver case surgical planning prototype using soft hydrogels. In this case, agarose was used, as it has better-matching liver tissue softness properties (using shore scale and viscosity measurements) than known 3D printing manufacturing strategies. Others can be used, such as polyvinyl alcohol/phytagel (PVA/PHY), as carried out by Forte et al. [24]. However, some of the models using hydrogels may start to degrade after 72–96 h, as they have important amounts of water that are progressively lost. Thus, polymeric- or silicon-based models represent a better alternative when it comes to material stability over time. For instance, silicone printing is becoming more popular and its use for anatomical models and implant production is increasing due to its ability to mimic soft tissue properties at similar ranges of elasticity as hydrogels, with higher biocompatibility and mechanical performance [45,46,47]. Most silicone or hydrogel models are produced by casting or molding. Molding and casting approaches are cost-effective, although they are time consuming, in terms of machinery and labor. Another possibility, as mentioned above, is the use of MJ technologies, since it is possible to achieve a wide range of softness values [45,48]. Unfortunately, this technology is not as affordable as molding or casting (indirect 3D printing). Nevertheless, the mentioned softness is only necessary in case the surgeons need to carry out hands-on training.

Table 6 summarizes the comparison of viscoelastic mechanical properties of the main tissues and materials involved in the present study. Finding specific data on viscoelastic properties such as shore hardness and elastic modulus for human tissue is challenging, as this specific measurement seems less commonly reported in the literature and can vary significantly based on a variety of physio-pathological and intra- and extra-cellular structural factors.

As explored in the present study and in the literature research [76], although different strategies (3D printing, molding, casting and injection) exist for the manufacture of anatomical models using synthetic materials (polymeric, resin, ceramic and others) and their use is expanding [77], in most cases, they still fail to provide the same haptic feedback as natural organs [78]. New materials such as hydrogels or silicones are the most promising options for mimicking soft tissues, as their elastic modulus and viscoelastic properties lie within similar ranges as those of natural tissues. For bone, with elastic modulus at the range of MPa for trabecular bone and GPa for cortical bone, current strategies are closer to realism, as significant research has been carried out in the fields of bone tissue engineering with metals and ceramics for reconstructive surgery, orthopedics, maxillofacial surgery and odontology. Thanks to advances in the field, bone has become the second most transplanted tissue globally [79]. In this field, the use of 3D bone scaffolds as extracellular matrixes providing an environment for cell adhesion, differentiation and growth has become a leading research trend. The abundance of materials includes metallic, ceramic and hybridized materials. For instance, Zhang et al. [80], in their review of the mechanical properties of materials for bone tissue engineering, found that between existing metallic options (stainless steel, titanium, magnesium or zinc), titanium alloys such as Ti-6Al-4V are widely used for bone reconstruction showing an elastic modulus of between 0.2 and 26.3 GPa. According to Zhang et al., this variation in the mechanical properties of the final parts and scaffolds is influenced considerably by the unit cells’ design and the inner architecture (pore size, morphology, etc.). It also stresses the need for graded functional characteristics in clinical applications to better mimic bone structures. On the other hand, Kanwar et al. [79] and Mirkhalaf et al. [81] review the existing synthetic bone alternatives to allografts and autografts, as they have the potential to improve biomechanical and biological properties. Many alternatives are explored, including ceramics, glasses, metals and polymers. Those alternatives include titanium dioxide, Bioglass 45S5; calcium phosphates such as hydroxyapatite and α- and β-tricalcium phosphate (α- and β-TCP); calcium silicates; and alternatives to the existing ceramics, with specific dopings, such as, zirconium (Zr), zinc (Zn), iron (Fe), silver (Ag), strontium (Sr), copper (Cu) and magnesium (Mg) among other composites. All the alternative doping strategies within their crystal structure allow for the improvement and design of tailored biological and mechanical properties. When it comes to 3D-printed scaffolds, bioceramics such as calcium silicates, polymers such as polycaprolactone (PCL) and composites such as PCL-hydroxyapatite are the norm [81]. PCL elastic modulus can be widely tuned from 27.3 ± 12.0 kPa to 1944.0 ± 228.7 kPa by adapting the internal architecture and design [82]. Jing et al. have higher values for PCL, with results around 5–7 MPa, increasing with the filling of hydroxyapatite as follows: 7–10 MPa (1%), 20–23 MPa (5%) [83]. Another alternative is bone cement such as Calcium Phosphate (CaP), whose properties can also be reinforced, as shown by Fada et al. [84], by tuning the internal porosity architecture and doping the material with nanoparticles such as strontium nitrate nanoparticles (NPs). The result is a material that can be tuned by modifying the porosity and filling in a range with a strength of between 10.3 MPa and 28.5 MPa, before and after placing the samples in simulated body fluid.

Metal bone-like scaffolds are normally preferred for load-bearing orthopedic and maxillofacial applications due to their greater toughness and damage tolerance. Ceramic, polymeric and doped composites are preferred for non-load-bearing applications due to their semi-controlled biodegradability and bone integration capacities, although they exhibit brittleness, making them fragile at load-field applications [80,81]. The former are preferred when it comes to simulation and training models, as they represent a cheaper but more robust alternative.

However, optimal replication of body tissue properties is defined by a complex combination of multiple anatomical components, combining complex tissue structures as well as liquids. For instance, most organs combine multiple core tissue types with supporting tissues (like extracellular matrix) with intra- and extra-cellular structural behaviors [85]. Oncological surgical planning models specifically need to reflect the physiological features of natural tissues, such as lymphatic node and vessel behaviors, tension lines, as well as oncotic/osmotic pressure of the tumor area. These are important factors associated with surgical planning, but cannot be reflected nowadays with the existing techniques.

For the moment, 3D printing can realistically replicate the geometric structures of anatomical models and can replicate multiple colors and textures, but without precision in mimicking the mechanical properties of soft tissues. This makes it a useful tool, depending on the level of realism required, for several educational and surgical planning purposes [86,87,88,89,90].

Additionally, the future availability of new hybrid multi-material 3D printers, combining technologies such as FFF and DIW (Direct Ink Writing, also a Material Extrusion technology) [91,92], looks promising. These new machines will allow the manufacture of advanced multi-material models without using a mold.

### 4.5. Comparison of Applications by AM Technologies

Table 7 summarizes each of the potential applications that could be carried out with each of the eight strategies presented in this work for the production of patient-specific anatomical models.

### 4.6. Summary and Future Perspectives

To summarize, from the eight different strategies considered (see Figure 3), (1), (2), (3), (4), and (7) can be good approaches for visualizing anatomical relations, patient–professional communication, and education. Meanwhile, (5), (6) and (8) represent better options for surgical planning and hands-on simulation and training. Each alternative presents different costs, times and advantages, as discussed, which can represent better options depending on the use and context.

Finally, the future perspective for the manufacture of the current prototypes would be through the development of hybrid multi-material medical-grade 3D printers. The FFF could be an affordable alternative for the hard parts, such as bone and less important anatomical structures; the softer parts, such as the soft tissues, could be manufactured using the DIW technique. However, until now, combining these two technologies has mainly only been established in proof-of-concepts.

### 4.7. Limitations of the Present Study

The limitations of the present study can be summarized in the following points: (1) the aim of the project was to show eight strategies based on real oncological pediatric abdominal cases. Thus, different cases are used and compared rather than using the same case with different technologies. Although they are not the same case, they all correspond to similar abdominal pediatric tumors (hepatic or neuroblastoma tumors) of similar dimensions and the calculation of cost and time is shown in ranges. (2) The mechanical viscoelastic properties of the materials used are compared with those of the natural tissue and organs. However, although a thorough search of the literature has been made, data regarding viscoelastic properties for human tissue are scarce and can vary significantly based on tissue structural factors and testing methods. (3) As reported, the synthetic models are unable to reflect important physiological factors associated with surgical planning and organ behavior, such as lymphatic node/vessels, tension lines, oncotic/osmotic pressure of the tumor area and the nature of tissue stiffness. This is due to the challenge of reflecting the anisotropic nature of natural tissue, as well as the effects of the interaction of different tissues and liquids in a living organ or tissue.

## 5. Conclusions

In the present work, eight different strategies for the manufacture of patient-specific surgical planning oncologic anatomical models have been presented and compared in terms of manufacturing time needed, cost per case, mechanical properties and potential applications. The following conclusions can be summarized from the manuscript:

- All the AM technologies and strategies presented can be used for the manufacture of 3D-printed models, but each one has both advantages and disadvantages. Thus, the decision on which strategy to choose will depend on clinical needs and available resources.
- Assuming access to all technologies presented and production capacity to reproduce the eight strategies of the present work, we conclude that if there is no need for hands-on training in a particular case, the best option may be FFF due to its simplicity in printing, multi-material printing capacity and accessibility in terms of costs.
- In case of complex surgery with hands-on planning and preparation needed, or in the case of surgical hands-on training and simulation in need of high accuracy models, MJ multi-material or indirect 3D printing should be used, as they allow for a combination of colors, hardness values and textures to provide a more realistic outcome.
- Among the different technologies, indirect 3DP is faster and cheaper in terms of material cost, but more expensive in terms of machinery, as it requires having two technologies available, and it needs trained personnel to dedicate significant time to the post-processing. MJ multi-material, on the other hand, requires less training and just one machine. For its part, MJ may be a better option for high-end, quasi-realistic surgical planning prototypes.
- Limitations exist in the presented and known strategies focusing on the production of models using synthetic materials (polymers, ceramics, etc.), especially for their inability to reflect important physiological features and the natural mechanical behavior of tissues. However, there are promising improvements in research using new technologies and materials based on hydrogels and silicones with advanced rheological and mechanical properties.

In summary, 3D printing and indirect printing technologies are promising tools for surgical planning, patient–professional communication and training in the treatment of abdominal pediatric oncology. Our work presented eight different strategies, all of them presenting advantages and limitations. Future work is needed to improve material properties and multi-material printing to achieve natural tissue physiological behavior.

## Figures and Tables

**Table 1:** List of materials and 3D technologies used in this study.

Material	AM Technology	Manufacturer	City and Country of Origin	Printer	Manufacturer	City and Country of Origin	Printing Software
Surgical Guide Resin	SLA	Formlabs	Massachusetts, USA	Form 3BL	Formlabs	Massachusetts, USA	PreForm 3.31.0
PLA	FFF	JF Polymers	Suzhou City, Jiangsu Province, China	Sigma R19/Customized FFF printer	BCN3D	Barcelona, Spain	Stratos 2.0.0/Simplify3D 4.1.0
PVA	FFF	JF Polymers	Suzhou City, Jiangsu Province, China	Customized FFF printer	BCN3D	Barcelona, Spain	Simplify3D 4.1.0
TPU (60A)	FFF	Recreus	Alicante, Spain	Customized FFF printer	BCN3D	Barcelona, Spain	Simplify3D 4.1.0
PA12	SLS	3D Systems	Hemel Hempstead, UK	Ricoh AM S5500P	RICOH	Tokyo, Japan	3DPrinterOS Ver.4.24.0.0
Vero White	MJ	Stratasys	Minnesota, USA	Connex 3	Stratasys	Minnesota, USA	GrabCAD 1.83
SUP706	MJ	Stratasys	Minnesota, USA	Connex 3	Stratasys	Minnesota, USA	GrabCAD 1.83
Vero Magenta	MJ	Stratasys	Minnesota, USA	J5 MediJet	Stratasys	Minnesota, USA	GrabCAD 1.83
Vero Cyan	MJ	Stratasys	Minnesota, USA	J5 MediJet	Stratasys	Minnesota, USA	GrabCAD 1.83
Elastico Clear	MJ	Stratasys	Minnesota, USA	J5 MediJet	Stratasys	Minnesota, USA	GrabCAD 1.83
SUP710	MJ	Stratasys	Minnesota, USA	J5 MediJet	Stratasys	Minnesota, USA	GrabCAD 1.83

**Table 2:** List of the 3D printing technology, material, design software and printing software used in each of the 8 strategies and models presented.

Strategy	Technology	Material	Design Software	Printing Software
1	SLA	Surgical Guide	Materialise MIMICS v.25	PreForm 3.31.0
2	FFF	PLA	Materialise MIMICS v.25	Stratos 2.0.0
3	Customized FFF printer	PLA-TPU-PVA	Materialise MIMICS v.25	Simplify3D 4.1.0
4	SLS	PA12	Materialise MIMICS v.25	3DPrinterOS 4.24.0.0
5	MJ Bi-material	Vero, SUP710	Materialise MIMICS v.25	GrabCAD 1.83
6	MJ Multi-material	Vero, SUP 706 and elastic resin	Materialise MIMICS v.25	GrabCAD 1.83
7	Hybridization of FFF + SLS	PA12—PLA—PVA	Materialise MIMICS v.25 and MeshMixer 3.5	Stratos 2.0.0 /3DPrinterOS 4.24.0.0
8	Indirect 3D Printing (casting)	PLA—TPU—PA12—silicone and hydrogel casting	Materialise MIMICS v.25 and MeshMixer 3.5	Stratos 2.0.0 /3DPrinterOS 4.24.0.0

**Table 4:** Machine, fungible and 3D printing material costs for the manufacture of 3D-printed surgical planning prototypes. * Cost can vary depending on the dimensions of the model.

Strategy (Figure 3)	Technology	Machine Cost (EUR RRP including VAT)	Fungible Material Cost [EUR/Time]	Machine Annual Maintenance Cost [EUR]	3D Printing Material Cost per Model * [EUR]
1	SLA	15,124	332.75/6 months	500	80–120
2	FFF	4229	80/4 months	200	50–80
3	Customized FFF printer	15,000	80/4 months	200	75–100
4	SLS	635,500	4000/5 years	2500	193–240
5	MJ Bi-material	29,800	1500/5 years	2125	480–600
6	MJ Multi-material	75,000	2000/5 years	2600	480–600
7	Hybridization of FFF + SLS	Combination of FFF and SLS	Combination of FFF and SLS	Combination of FFF and SLS	350–400
8	Indirect 3D Printing (casting)	Combination of FFF and SLS	Combination of FFF and SLS	Combination of FFF and SLS	156–200

**Table 5:** Comparison of layer thickness resolution of each technology used in the present study. All numbers are publicly disclosed by each manufacturer.

Technology	Layer Resolution (Layer Heights) (µm)
SLA	25–100
FFF	50–400
SLS	80–120
Indirect 3D Printing	50–400
MJ Bi-material	28
MJ Multi-Material	18

**Table 6:** List of materials and 3D technologies used in this study and their main viscoelastic mechanical properties (shore hardness and elastic modulus).

Material	Technology	Manufacturer	Printer	Manufacturer	Shore Hardness	Elastic Modulus	Methods
Surgical Guide	SLA	Formlabs	Form 3BL	Formlabs	67 (D)	2900 ± 90 MPa	ASTM D790 [49], ASTM D638-10 [50]
PLA	FFF	JF Polymers	Sigma R19/Customized FFF printer	BCN3D	76.8 (D)	1568 ± 45 MPa	ISO 527-2/5A/50 [51], ISO 178 [52], ISO 868 [53]
TPU (60A)	FFF	Recreus	Customized FFF printer	BCN3D	63 (A)	26 ± 5 MPa	DIN ISO 7619-1 [54], DIN 53504-S2 [55]
PA12	SLS	3D Systems	Ricoh AM S5500P	RICOH	73 (D)	1487 ± 48 MPa	ASTM D638, ASTM D790, ASTM D2240 [56]
Vero White	MJ	Stratasys	Connex 3	Stratasys	86 (D)	1492 ± 175 MPa	ASTM D638-03-04-05, D790-04, DMA E
SUP706	MJ	Stratasys	Connex 3	Stratasys	86 (D)	1492 ± 175 MPa	ASTM D638-03-04-05, D790-04, DMA E
Vero Magenta	MJ	Stratasys	J5 MediJet	Stratasys	86 (D)	1492 ± 175 MPa	ASTM D638-03-04-05, D790-04, DMA E
Vero Cyan	MJ	Stratasys	J5 MediJet	Stratasys	86 (D)	1492 ± 175 MPa	ASTM D638-03-04-05, D790-04, DMA E
Elastico Clear	MJ	Stratasys	J5 MediJet	Stratasys	45 (A)	4 ± 2 MPa	ASTM D412 [57], ASTM D395 [58], ASTM D2240
Agarose hydrogel	Indirect 3D Printing (casting)	Químics Dalmau, Spain	-	-	17 (00)	5.5 ± 3.1 kPa	ASTM D2240
Silicone	Indirect 3D Printing (casting)	Dragon Skin^®^	-	-	4 (00)	38.8 ± 18.7 kPa	ASTM D2240
**Tissue**	**References**				**Shore Hardness**	**Elastic Modulus**	**Methods**
Liver	Estermann et al. (2020) [59], Yoon et al. (2017) [60], Tejo-Otero et al. [25], Forte et al. [24]				13–30 (00)	1.4 ± 0.8 kPa–5.49 ± 1.2 kPa	ASTM D2240
Kidney	Kaiyan et al. (2018) [61], Tejo-Otero et al. [32], Amador et al. (2011) [62]				28–40 (00)	4 ± 1.8 kPa–17 ± 2.5 kPa	ASTM D2240
Vessels	Arm R, et al. (2022) [63], Camasão et al. (2021) [64], Zhang et al. (2005) [65]				40–45 (00)	300–600 kPa	ASTM D2240
Tumor	Monferrer et al. (2020) [66], Tejo-Otero et al. [7], Kawano et al. (2015) [67]				30 (0)–22 (A)	0.58–45 KPa	ASTM D2240, and various experimental set-ups
CorticalBone	Kurtz et al. (2023) [68], Keaveny et al. (1993) [69], Zysset et al. (1999) [70],				-	7–35 GPa	ASTM D2240 and various experimental set-ups
Trabecular bone	Yoon et al. (2021) [71], Lefèvre et al. (2019) [72], Morgan et al. (2018) [73]				-	10–3000 MPa	Various experimental set-ups
Bone marrow	Wang et al. (2022) [74], Jansen et al. (2015) [75]				-	0.25–24.7 KPa	Various experimental set-ups

**Table 7:** Comparison of potential application per each of the eight presented strategies. MM is multi-material. 3DP is 3D printing. √ means good use. √√ means excellent use.

Use	SLA	FFF	FFF MM	SLS	FFF + SLS	Indirect 3DP	MJ	MJ MM
Visualize anatomical relationships	√	√	√	√	√√	√√	√	√√
Pre-surgical planning and adaptation of implants	√	√	√	√	√	√	√	√√
Patient–professional communication	√	√	√√	√	√√	√√	√	√√
Simple simulation	√		√		√	√	√	√
Hands-on training						√	√	√√

## Data Availability

Data are unavailable due to privacy or ethical restrictions.
